# Comparing priority received by global health issues: a measurement framework applied to tuberculosis, malaria, diarrhoeal diseases and dengue fever

**DOI:** 10.1136/bmjgh-2023-014884

**Published:** 2024-07-08

**Authors:** Rakesh Parashar, Sharmishtha Nanda, Stephanie L Smith, Zubin Shroff, Yusra R Shawar, Dereck L Hamunakwadi, Jeremy Shiffman

**Affiliations:** 1 Health Systems and Policy, Independent Consultant, New Delhi, Delhi, India; 2 Global Business School of Health, Faculty of Population Health Sciences, University College London, London, UK; 3 Independent Consultant, Delhi, India; 4 International Center for Research on Women Asia Regional Office, New Delhi, India; 5 School of Public and International Affairs, Virginia Tech, Arlington, Virginia, USA; 6 WHO Alliance for Health Policy and Systems Research, Geneva, Switzerland; 7 International Health, Johns Hopkins University, Baltimore, Maryland, USA; 8 Virginia Tech Research Center–Arlington, Arlington, Virginia, USA

**Keywords:** Global Health, Health policy, Health systems, Tuberculosis, Malaria

## Abstract

**Introduction:**

The relative priority received by issues in global health agendas is subjected to impressionistic claims in the absence of objective methods of assessment of priority. To build an approach for conducting structured assessments of comparative priority health issues receive, we expand the public arenas model (2021) and offer a framework for future assessments of health issue priority in global and national health agendas.

**Methods:**

We aimed to develop a more comprehensive set of measures for conducting multiyear priority comparisons of health issues in six agenda-setting arenas by identifying possible measures and data sources, selecting indicators based on feasibility and comparability of measures and gathering the data on selected indicators. We applied these measures to four communicable diseases—tuberculosis (TB), malaria, diarrhoeal diseases and dengue fever—given their differing impressionistic claims of priority. Where possible, we analysed the annual and/or 5-year trends from 2000 through 2022.

**Results:**

We observed that TB and malaria received the highest priority for most periods in the past two decades in most arenas. However, a stagnation in development funding for these two conditions over the last 8–10 years may have fuelled the neglect claims. Despite having a higher disease burden, diarrhoea has been slipping in global priority with reduced spending, fewer clinical trials and stagnating publications. Dengue remains a low-priority condition but has witnessed a sharp rise in attention from the pharmaceutical industry.

**Discussions:**

We expanded the arenas model by including a transnational arena (international representation) and additional measurements for various arenas. This analysis presents an approach to enable comparative trend analysis of the markers of agenda status over a multiyear period. More such analyses can bring much-desired objectivity in understanding how attention to global or national health issues changes over time in different arenas, potentiating a more equitable allocation of resources.

**WHAT IS ALREADY KNOWN ON THIS TOPIC**

- Approaches to understanding the comparative priority received by health issues in global and national health agendas are in an early stage of development.
- The public arenas model offers a conceptual entry point to measure health issue priority in global health agendas.

**WHAT THIS STUDY ADDS**

- This paper adds to the body of work that advances systematic measurement of issue prioritisation in global health agendas.
- Our analysis shows that priority and resource allocation for health issues differ between arenas, vary at different points in time and only occasionally follow the burden of disease patterns.

**HOW THIS STUDY MIGHT AFFECT RESEARCH, PRACTICE OR POLICY**

- The set of six transnational arenas and measures of priority proposed in this work can be useful for policymakers and researchers to identify specific priority given to health issues by various types of actors and allow for a more just allocation of resources.

## Introduction

 The priority status of health issues in global or national contexts is often contested as several claims are made about which issues receive priority and which face neglect.^1^ Several factors may shape these impressionistic claims (claims which are not substantiated with valid data), such as the positions and affiliations of the actors who make these claims, the influence of popular discourse in the media, the perceived outlook and priorities of country governments and other global health agencies, fuzzy assessments of funding, public sentiments, development goals and other potential markers of policy attention. Advocates often use such claims to gain attention and resources among shifting global priorities. While there is a rich and growing scholarship about why and how certain health issues receive attention at global and national levels, the tools to measure the differential status of health issues in the global health agenda are not well developed, and it is not entirely possible to validate the claims of priority or neglect.^1^,^6^ The absence of approaches to compare claims with measurable indicators could contribute to the inappropriate and unfair distribution of resources. A systematic approach is needed to validate priority claims and establish the agenda status of health issues more objectively.^6 7^

To move from impressionistic claims related to the priority received by global health issues to their systematic assessment, Smith and colleagues^1^ proposed the public arenas model—as a novel means of addressing conceptual and measurement challenges in understanding the priority received by global health issues.^3 8 9^ The paper offers a cross-sectional view of the agenda status of the studied conditions by applying an initial set of measurements during the event of the COVID-19 pandemic. The authors focused on five transnational arenas for global health agenda setting, including international aid, industry, scientific research, news media and civil society. Each of the five arenas was measured using status indicators, such as development assistance for health (DAH) for international aid, drug trial registrations for the pharmaceutical industry, bibliographic trends in medical and public health journals for scientific research, media trends such as column inches and minutes of air time devoted to each issue for news media, and civil society mobilisation on selected issues for the civil society arena.

In the current paper, under the aegis of the same project—the Global Health Agendas Project (GHAP)—we aimed to expand the applicability of the arenas model and develop a detailed set of arenas and measurements that can be used to analyse the priority of health issues over longer periods and in comparison, with each other, globally and potentially in country contexts. Our definition of arenas is informed by the work of Hilgartner and Bosk,^8^ who point to social action groups, research communities, religious organisations, news and entertainment media, professional societies and branches of government as public arenas in which social problems are defined and resources are allocated.^1 8^

To develop the expanded set of measurements and apply them, we selected four health issues: tuberculosis (TB), malaria, diarrhoeal diseases (DD, henceforth diarrhoea) and dengue fever (henceforth dengue). We have presented the reasons for choosing these conditions in the next section of the paper. Our analysis aimed to objectively ascertain which of the selected conditions received relative priority or neglect in key agenda-setting arenas, during which periods of time and whether the agenda status was consistent with the burden of disease of these issues at that time.

The work presented in this paper contributes to the scholarship on measuring the agenda status in two main ways. First, we expand the transnational arenas and measurements and conceptualise a new arena and measurements to allow for future global or country-level analyses. Second, we provide a comparative trend analysis of the markers of agenda status over 20 years and compare these with the burden of disease in the relevant periods, the first such longitudinal analysis. Another paper^10^ further supplements the work presented in this paper, which analyses the trends in priorities received by disease conditions in relation to the policy and agenda-setting theories and adds to theory building in this area.

### Reasons for selecting TB, malaria, DD and dengue

We selected these diseases for our analysis as they make an interesting set of infectious diseases, offering an opportunity for comparability in their disease burden and claims of priority in global health agendas. All four conditions are part of the stated global health priorities reflected in the Sustainable Development Goal (SDG) targets. There are specific targets under the SDGs for ending TB, malaria and neglected tropical disease (NTD, including dengue) epidemics by 2030 (specifically SDGs 3.3.2, 3.3.3, 3.3.5 and 3.9.2). While diarrhoea does not have a specific SDG progress indicator despite being considered essential for achieving the SDG target of under-5 mortality (SDG 3.2.1), it is included under indicator 3.9.2 (substantially reduce the number of deaths and illnesses from hazardous chemicals and air, water, and soil pollution and contamination). However, the priority received by these conditions is understood differently by various actors, given the varying claims and inconsistent evidence on both prioritisation and neglect.^11^ Both TB and malaria are said to have received a high degree of attention (along with HIV/AIDS) in the past two decades, giving rise to claims of neglect of diarrhoea and other infectious diseases not supported by global partnerships such as The Global Fund to Fight AIDS, Tuberculosis and Malaria and the STOP TB Partnership. However, we also noted claims of neglect around TB and malaria in general and more frequently during the COVID-19 pandemic,^12^,^17^ which are contrary to other claims and make these conditions interesting cases to assess their agenda status objectively. Diarrhoea has been discussed as a slipping priority in global health despite its continued high occurrence and being one of the top causes of childhood mortality. Likewise, the group of conditions listed under the NTDs is considered ‘neglected’ in the global health priorities, the very reason NTDs were given their name.^2 18^ Dengue is one of the leading contributors to the disease burden among the NTDs, and several actors have demanded greater attention for dengue^19^ in the global health agenda, which may have shifted the priority being received by dengue.

These conditions also represent high-burden and low-burden communicable diseases. TB, malaria and diarrhoea are some of the leading contributors to global disability-adjusted life years (DALYs) linked to infectious diseases, while dengue has been rising in its burden.^11^ The Global Burden of Disease (GBD) estimates^20^ show that diarrhoea is the highest burden condition among these four for the full study period of 2000–2022, followed by TB, malaria and dengue (online supplemental figure 1). In 2019, DALYs (per 100 000 population) were 20.95 for diarrhoea, 14.65 for malaria and 8.96 for TB—the same order as by mortality burden. While there has been a downtrend for TB, malaria and diarrhoea over this time period, the rate of decline for DALYs has been relatively slower for malaria, with stagnation in decline observed between 2014 and 2019. While the burden of disease and deaths for TB, malaria and diarrhoea has gone down since 2000, the burden of dengue has been increasing, with dengue being one of the fastest rising vectorborne diseases, putting almost 50% of the world population at risk.^21^ The GBD data trends between 2000 and 2019 show that the incidence, death rates and DALYs all increased for dengue in this period, with the burden of deaths and DALYs shifting to the adult population compared with the earlier higher burden in the under 5 years age group. The global burden of dengue is also different from the other three conditions as most of the dengue cases are concentrated in Southeast Asia and have been rising in endemic areas in the Americas.

In the next sections, we present the approach about how we conceptualised the arenas and measurements, offer an analysis of trends in the markers of agenda status for the identified health conditions for over 20 years and reflect on the agenda status of these conditions across arenas. We conclude with a framework that enlists each arena’s indicators and their data sources, which can be used for future global or country-level analyses.

## Methods

In this section, we describe the methodology through which we built on the arenas and measurements suggested in the public arenas model, identified new arenas and identified appropriate indicators to measure priority in these arenas. We then provide information on how we collected data to help measure these indicators and how we came up with a final selection of indicators.

### Conceptualising public arenas and identifying measurements

We began by reviewing the five transnational arenas proposed in the earlier paper^1^: international aid, news media, the pharmaceutical industry, scientific research and civil society. In addition, we also reviewed theoretical approaches proposed by scholars to understand the scope for expanding the public arenas applicable to health agenda setting. We introduced a transnational arena in the paper—the ‘International Representation Arena’,^22^ which was conceptualised as *interstate organisations that deal with global and cross-border health issues and provide technical guidance, policy, planning and response support for these issues, and in turn allocate resources to health issues and influence their agenda priority*. We conceptualise this arena as a critical addition to the public arenas model for measuring health agenda setting as it includes multiple influential actors, including interstate organisations (such as the United Nations and the WHO), multilateral organisations (such as the World Bank), international non-governmental organisations, as well as social activists and movements, and represents a cohesive set of interests and actions on matters of global health. However, potentially, more arenas can exist within these groups of actors, and there would be several interactions and overlaps across these arenas. Broadly, we acknowledge that most agenda-setting arenas would have interactions and overlaps, and can influence each other.

We then conducted a literature and database search to identify an exhaustive set of measurements for all six arenas. We did open Google searches and searched the potential databases (this exercise was not a systematic search or review) with a combination of keywords applicable to potential indicators for each arena. For example, for the international aid arena, we used terms such as DAH and global spending on health issues or diseases, among others. At this stage, we included numerical and narrative measures for existing and proposed arenas. Second, we listed potential indicators for each measure and sources for capturing data and/or information against each indicator. This process provided us with a broader set of measurements, which may or may not apply to all health conditions, all arenas and all country contexts. For example, for the international aid arena, we initially identified DAH, inclusion in aid priority strategies, availability of aid impact data and availability of air effectiveness studies as potential measures. We identified numerical indicators for the first two measures and narrative indicators for the last two measures, respectively. This included US dollars spent (for measuring DAH), the number of strategic aid documents pledging commitments to selected disease conditions (for inclusion in aid priority strategies), analytical insights from outcome/impact narratives related to the aid for a particular disease (for aid impact data) and availability and quality of cost-effectiveness data linked to aid (for aid effectiveness measure). After this preliminary search and exhaustive listing of arenas, measurements, indicators and data sources, we screened and selected a final set of arenas and measurements from a validity, feasibility and comparability point of view as presented ahead (online supplemental table). Taking the example shared above, we only selected DAH as a measure to include in the final analysis.

### Data collection and selection of indicators

After listing various measures based on the literature review, we attempted to collect data for all listed indicators from 2000 to 2022. For databases with a monthly date selection, we set the start date to 1 January 2000 and the end date to the day of data collection (this ranged from October to December 2022). For some indicators, data were available only until 2020 or 2021, which we considered the end year. Since the paper’s focus is to examine the long-term patterns of change, the unavailability of the last 1-year or 2-year data for these indicators did not change our interpretations. For each indicator, the search terms and spellings were kept consistent, which were malaria, TB, diarrhea and diarrhoea, and dengue. Variations for diarrhoea and dengue included *newborn and child health diseases* and *neglected tropical diseases* depending on the databases.

In the first round of data collection, we tested the feasibility of using the listed indicators and decided to exclude any indicator that did not have numerical or narrative data sources that were easily accessible for selected diseases. Where data were available, we checked for construct validity, that is, whether the measurement was appropriate to measure the construct in question. The authors’ consensus was used here because existing subject knowledge can be used to judge construct validity. Next, we looked for comparability, that is, data were available in a standardised or comparable format at the global level and over time. Data transparency (with most accessible without a paywall) was also considered while screening for sources. It is important to note that all the indicators selected after this process were retained primarily because they fulfilled all or most of the selection criteria. Once we selected the above arenas and indicators, we analysed trends within each arena and presented the results for at least one measure in each arena. Due to differences between arenas and corresponding indicators, the data collection approaches for each arena vary and are summarised in box 1.

Box 1 **Methods, measures and indicators for each arena**

**International aid arena:** We collect and analyse allocations and spending from the development assistance for health (DAH) figures in US dollars by health focus areas from 2000 to 2020, using the data tracked by the Institute for Health Metrics and Evaluation. We maintain category names from the database for tuberculosis and malaria, for which direct tracking is available, and we track the DAH trends for diarrhoea using the category name ‘Newborn and Child Health-other’* and trends for dengue using the category name ‘Other Infectious Diseases-other’**. We maintain category names from the database, particularly ‘Other infectious diseases-other’ and ‘Newborn and Child Health-other’ for specificity and to facilitate cross-reference with the database.

We acknowledge that the compiled information in the ‘other’ categories is not likely a robust proxy measure for dengue and diarrhoea because of multiple potential variations in the overall infectious disease and newborn health allocations. Still, we included these indicators because more specific information may be available in some country contexts, making this a valid measure for local analyses.

**Pharmaceutical industry arena:** Registered clinical trial data from the widely used ClinicalTrials.gov (sponsored by the US government) were searched using the following terms: *Tuberculosis, Malaria, Diarrhoea* and *Dengue*. Clinical trials sponsored by industry were filtered from the total clinical trials registered during the period 1 January 2000 to 31 December 2022.

**Scientific research arena:** Data on bibliographic entries were obtained using searches of four databases using the keywords *Tuberculosis, Malaria, Diarrhoea* and *Dengue* from 2000 to 2022. Specific search start and end dates were used where possible. All entry types were included. In addition, we included non-industry-sponsored trials as part of the scientific research arena using ClinicalTrials.gov as well as the WHO Trials Registry. We also used PROSPERO to look for the number of systematic reviews registered for the selected diseases in the desired duration. Search terms and criteria remained like the above. Lastly, we counted the number of series publications commissioned by *The Lancet* to indicate agenda status in the scientific research arena. As no advanced search tool was available here, this search was not specific to the time duration of 2000–2022, and the number of series per disease was hand-searched from the list of all *Lancet* Series publications.

**News media arena:** The annual worldwide number of news items was collected for the four keywords from the Access World News Archive, Google News Archive and Vanderbilt Television News Archive (2000–2021). We included Google search trends as an additional measure under the news media arena. We included ‘Google search trends’ despite not fully fitting into our conceptualisation of arenas, as the trends in internet searches may relate to the public interest and may indirectly influence news media and agenda priorities. We included Google search trends, signalling the total number of queries for each keyword for each year starting 2004 (as data prior to 2004 were not available) until 2021. However, we have presented the results only from the Access World News Archive as an illustration of the data from this arena.

**International representation arena:** In this arena, the key indicators were attention to issues in resolutions endorsed by members of the United Nations General Assembly (UNGA) and by the World Health Assembly (WHA). Google searches were conducted on 27 February 2023 using the terms UNGA resolution AND: (1) dengue, NTD, OR neglected tropical disease; (2) diarrhoea, water, sanitation, hygiene, OR WASH; (3) malaria; (4) tuberculosis. We reviewed the titles of all WHA resolutions (494) endorsed between 2000 and 2021, then used content analysis to identify 29 resolutions focusing on dengue, NTDs, diarrhoeal diseases (DD), safe WASH, malaria and tuberculosis.

We also collected data on key progress on Sustainable Development Goal (SDG) indicators linked to four selected disease conditions, which are as follows:

- Indicator 3.3.2: tuberculosis incidence (per 100 000 population).
- Indicator 3.3.3: malaria incidence per 1000 population at risk (per 1000 population).
- Indicator 3.3.5: number of people requiring interventions against neglected tropical diseases (number) **(as a proxy indicator for dengue)**.
- Indicator 3.9.2: mortality rate attributed to exposure to unsafe WASH services (per 100 000 population) **(as a proxy indicator for diarrhoea)**.

**Civil society arena:** We started with reviewing the websites of 217 civil society organisations that were among non-state actors holding consultative status with the WHO in 2020. We used the Wayback Machine (a digital archive of content on the internet accessed at https://web.archive.org/) to thematically analyse the contents of home and main program web pages dating to 2018, the last year during which sites of the organisations were consistently archived. 11 of the initial 217 organisations were eliminated from the analysis (n=6 websites defunct; n=6 archives incomplete).

^*^The category ‘Newborn and Child Health’ accounts for nutrition, immunisation, human resources, health systems strengthening and other. We have taken the figures for ‘other’ as it includes diarrhoea and offers a closer approximation to DAH on diarrhoea than the remaining categories.^**^The category ‘Other Infectious Diseases’ accounts for antimicrobial resistance, COVID-19, Ebola, human resources, health systems strengthening, Zika and other. We have taken the figures for ‘other’ as it includes dengue and offers a closer approximation to DAH on dengue than the remaining categories.

### Patient and public involvement

This study had no public or patient involvement, and all the data were based on anonymous secondary information.

Data collected against each indicator were mapped for all the indicators for all the years for which data were collected in separate sheets for each arena. Once the data were cleaned and organised, trends emerging for each indicator over time were plotted on graphs.

## Results

In this section, we present an analysis of data gathered for the studied health issues across the arenas based on the trends in the selected indicators. We have presented the results from at least one measure under each of the six arenas. We referred to GBD estimates between 2000 and 2019 (as presented in the Introduction section and in online supplemental figure 1) to compare the priority received by the diseases studied in various arenas.

### International aid arena

**Figure 1:**
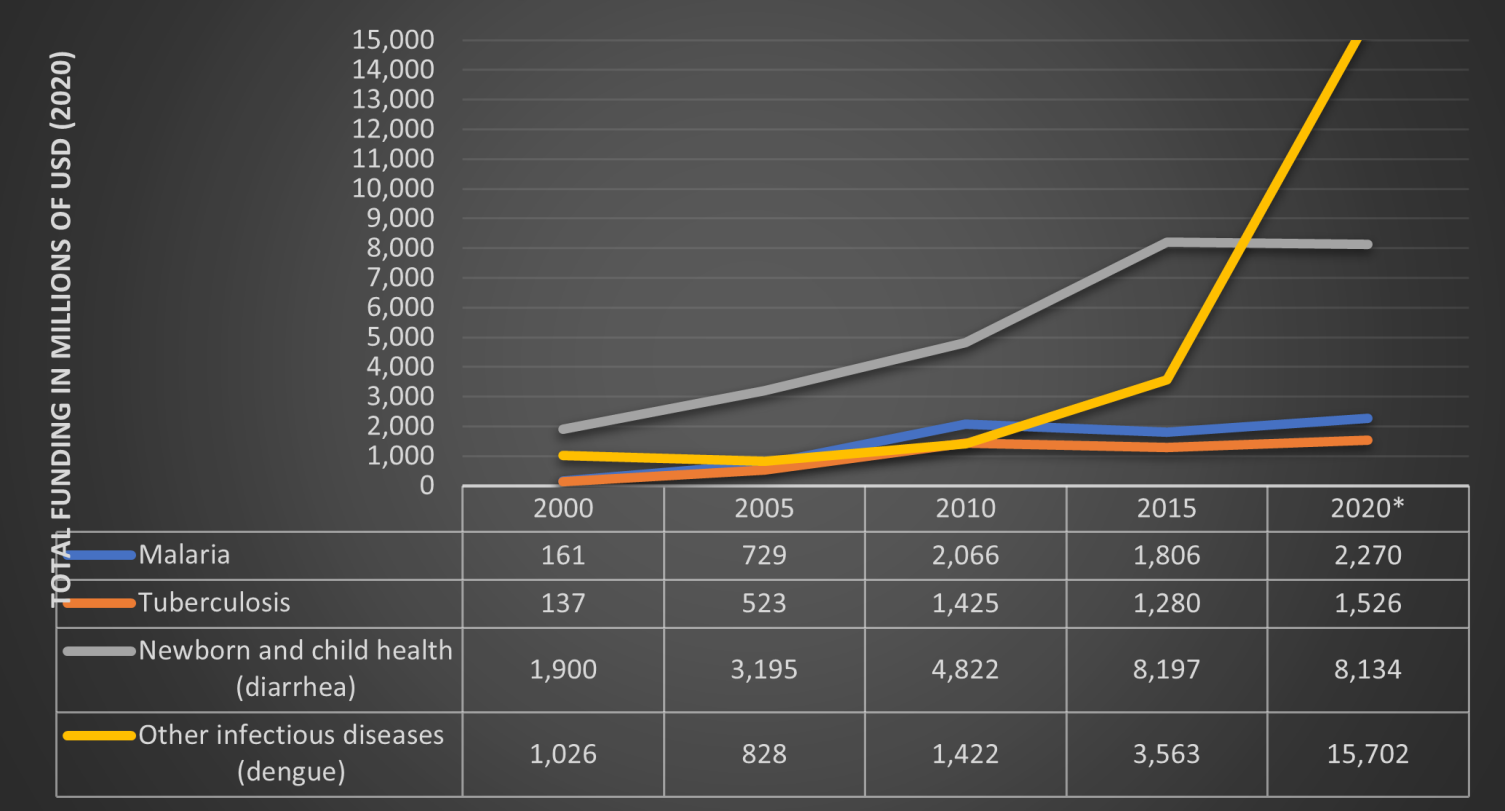
Five yearly development assistance for health (DAH) trends by health focus area in millions of US dollars (2000–2020).

As shown in 50 yearly break-ups of trends for DAH (figure 1), we noted that the DAH for malaria was higher than TB for the entire study period and gradually increased from 2000 to about 2010. The DAH for malaria and TB stood at US$160 million and US$137 million in 2000, which rose to about $2 billion and $1.425 billion by 2010. The DAH for malaria and TB grew only by 10% between 2010 and 2020. Also, the proportion of funding for these two conditions from the total DAH for all conditions shrank consistently. This trend continued until 2020, clearly showing more attention and resources going into areas such as health systems strengthening, reproductive health and newborn and child health, which may be reflected in the claims of neglect for TB and malaria. The proxy data for dengue (the ‘other’ category of DAH for ‘other infectious diseases’) showed the maximum rise in DAH for this category between 2005 and 2015 (growing from $763 million to $1.746 billion). However, after 2015, the DAH for this category also stagnated. For diarrhoea, the closest proxy indicator for DAH (the ‘other’ category within the Newborn and Child Health category) grew from $776 million to $2.194 billion between 2000 and 2015. However, after 2015, the DAH in this category also fell and was about $1.280 billion in 2020.

### Scientific research arena

The data from the WHO registry for clinical trials showed the highest number of trials registered for TB (2181), followed by malaria (1945), diarrhoea (1274) and dengue (1274). The data from ClinicalTrials.gov (online supplemental figure 2) additionally offered the number of non-industry clinical trials, suggesting the highest non-industry priority for TB and malaria (above 80% trials), which may relate to development assistance for these conditions or public funding from country governments and a lesser share of industry funding in scientific research. The non-industry trials were about 61% diarrhoea for and 51% for dengue. Likewise, the annual trends in the total registered trials showed that the number of annual trials has consistently increased for TB since 2000, while the numbers started stagnating or dropping for diarrhoea after 2010 and for malaria after 2015. There was a consistent dip in the total number of trials for all conditions during 2019–2020, which picked up again during 2021, showing a potential impact of COVID-19.

**Figure 2:**
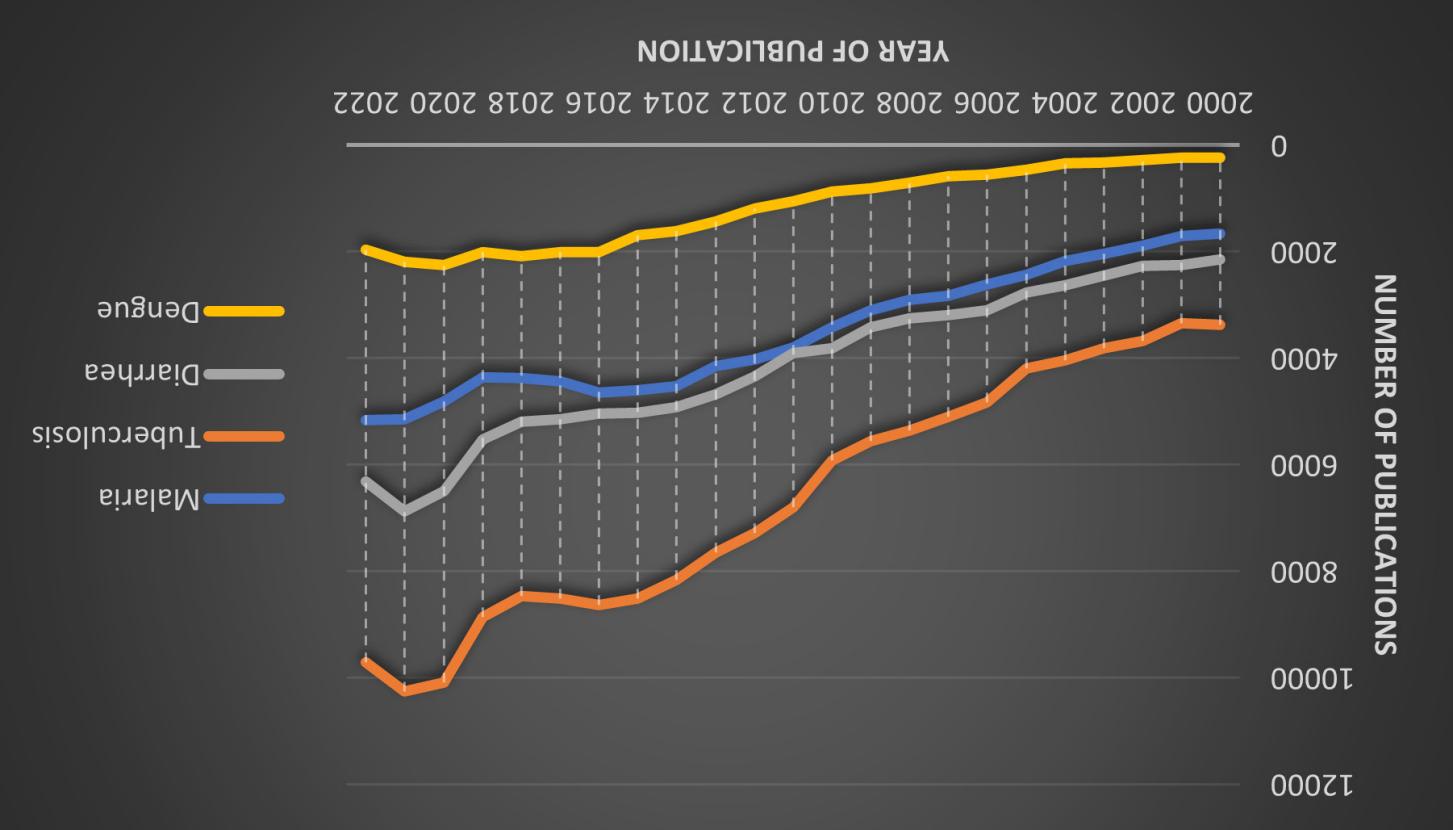
Yearly publication data by disease from PubMed (2000–2022).

As seen by the number of publications in the PubMed database (figure 2), the bibliographic trends indicate the scientific community has given relatively greater attention to TB, followed by diarrhoea and malaria. Dengue was part of the least number of publications. The annual trends of publications show a continued rise in publications for all conditions, with a relatively faster increase between 2010 and 2015 and stagnation in growth after 2015. *Lancet* Series publications were also found to be the highest for TB (12 series), followed by malaria (3 series), diarrhoea (1 series) and dengue (1 series). However, overall, NTDs have more series published compared with only dengue.

### Pharmaceutical industry arena

The total number of industry-sponsored clinical trials during the period of 2000–2020 was the highest for diarrhoea (359), followed by malaria (253), TB (189) and dengue (111). An analysis of the 5-year trends of the industry trials showed a continued rise in the industry trials for dengue, jumping from four trials from 2000 to 2005 to 40 trials during 2016–2020. The industry trials for TB, malaria and diarrhoea peaked between 2006 and 2015 and showed a downtrend afterwards. From 2016 onwards, diarrhoea had the sharpest reduction in the number of industry-sponsored trials, reducing by about 30% from the preceding 5-year period.

### News media arena

**Figure 3:**
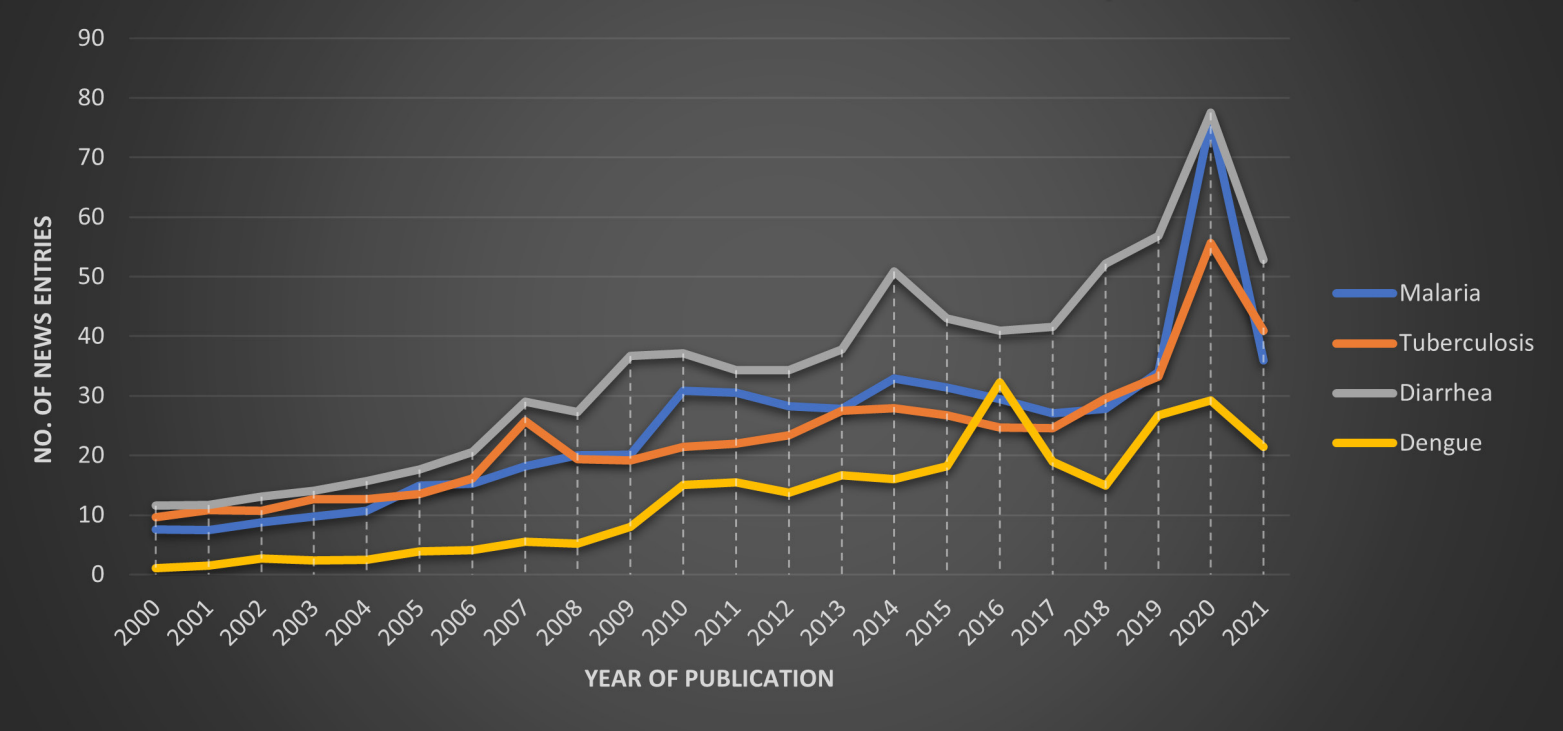
Annual news trends by diseases, Access World News Archive (2000–2021).

We gathered the data to represent the selected conditions in the published news from the Access World News Archive. Figure 3 shows that the mentions of diarrhoea were the highest throughout the study period, followed by malaria, TB and dengue in that order. Dengue has shown occasional spikes in its mention and was mentioned nearly twice as frequently as TB and malaria in 2015. Overall, the news media publishing on ‘health’ in 2021 was nearly 17 times compared with 2000 (2 204 069 articles compared with 131 773 articles). In 2020, the new coverage for all four conditions spiked, signalling potential higher coverage for any health issues because of overall heightened global attention to health linked to COVID-19.

### International representation arena

We found 47 United Nations General Assembly (UNGA) resolutions linked to the studied health issues between 2000 and 2021 (online supplemental figure 3). Malaria (24 resolutions) emerged as the priority leader, possibly because it was a focal issue in the United Nations Millennium Declaration (2000, setting up the Millenium Development Goals) and the 2030 Agenda for Sustainable Development (2015, introducing the Sustainable Development Goals). TB is also specifically addressed in the latter, but not the former, of these powerful norming resolutions. Overall, the UNGA endorsed six resolutions directly addressing TB during the study period, including three in 2014 and one from a high-level meeting on the fight against TB in 2017. Neither dengue nor diarrhoea is named in the title of any UNGA resolution during the study period. Both are covered by the 2030 Agenda for Sustainable Development resolution—under SDG 3 for health and well-being, dengue is among the major NTDs (SDG target 3.3.5) and diarrhoea among waterborne diseases of concern (SDG 3.3, no target). However, diarrhoea is also addressed indirectly by SDG 6—ensure the availability and sustainable management of (safe) water and sanitation for all. DDs are among the major health-related drivers of 22 resolutions aimed at improving access to clean water, sanitation and hygiene (WASH) throughout the study period.

From the 494 World Health Assembly resolutions in the study period, we identified 29 directly or indirectly addressing these issues. TB^15^ and malaria^13^ lead the pack, and six resolutions directly address diarrhoea. A 2002 resolution to prevent and control dengue fever and dengue haemorrhagic fever is the only one to address that problem directly; it is indirectly addressed in five NTD-focused resolutions.

We also analysed the progress against the SDG targets for TB (SDG 3.3.2) and malaria (SDG 3.3.3). Separate data for SDG indicator progress were unavailable for dengue and diarrhoea. Hence, we used the SDG targets 3.3.5 and 3.3.9 as proxy indicators for these two, respectively. We observed that the incidence of malaria and TB consistently declined for the analysis period, but the reduction in malaria incidence slowed down after 2013 and remained like 2014, in 2020. Between 2000 and 2020, the incidence of TB reduced from 174 to 127 per 100 000 people, and malaria incidence declined from 81 to 58 per 1000 people. From the point of view of dengue being recognised as one of the NTDs currently, it is also important to note that the number of people (in billions) who require treatment for any NTDs went down after 2012 but stagnated after 2016, as seen from the SDG indicators data (SDG 3.3.5). The overall slow reduction and stagnation in NTD incidence could be related to the rising incidence of dengue, as dengue contributes to a high proportion of incidence among NTDs.^23^

### Civil society arena

The data for this arena were curtailed from 2018 to 2021 due to challenges in accessing long-term and geographically representative data. We reviewed and coded the websites of 206 civil society actors for programmes addressing malaria, TB, dengue (or NTDs) and DD (or WASH). Analysis of 206 civil society organisations (CSO) websites for programme attention to the diseases shows TB leading malaria in 2018 and 2019 and the issues on par with each other in 2020 and 2021 (n=19 CSOs with programmes each) DD and then dengue trailed consistently. DD peaked in 2021 with 10 CSO websites showing programme priority, while dengue peaked in 2019 with programme priority from two CSOs (online supplemental figure 4).

## Discussion

Our efforts to develop a standardised or structured approach to understand what is prioritised or neglected in global health add to the small body of scholarship on measuring the priority status of health issues. This paper builds on the public arenas model^1 8^ and contributes to the scholarship on measuring the agenda status in two important ways. First, we offer an expanded set of arenas and measurements for assessing the priority of health issues at the global and country levels. We identified international representation an important arena for global health agenda setting, which mediates health issue prioritisation by several overlapping actions including setting up of development goals, health issue-specific commitments and roadmaps, global and regional health issue target setting, knowledge production, actor coordination and treaties among others. Second, for the first time, we provide a comparative trend analysis of the markers of agenda status over longer periods of time, which can allow an understanding of the issue priority within and across arenas during any specific time frame.

Applying the identified set of arenas and measures of priority within six global health arenas, we observed that TB and malaria have received the highest priority across all the arenas for most of the study years, with relative priority for TB and malaria varying across arenas. Diarrhoea, despite being the largest contributor to DALYs and deaths among the studied conditions, has received less priority compared with these two, with attention to diarrhoea further going down after around 2015, as seen in reducing development spending, reducing number of trials and publications and the absence of a dedicated SDG indicator. TB, being the second lead in the disease burden, has received the greatest attention across most arenas as reflected in higher total spending, continued and rising interest of the scientific community, relatively better progress on SDG targets (when compared with malaria) and higher coverage from the news media. However, the overall international aid for TB has only marginally increased since 2010, and industry-sponsored trials for TB have reduced, which may confirm the claims of neglect for TB in recent years. Malaria, third in the disease burden, has received more attention than diarrhoea. However, this attention has relatively gone down after around 2010, which can be observed by stagnated international aid since 2010, and slow progress against the SDG target for malaria in recent years. Dengue is low in priority overall but gradually rising on the agenda, especially gaining attention from the pharmaceutical industry, although it remains far behind compared with the other three conditions. Although a higher share of industry funding for scientific research for dengue may be specific to certain countries and may be linked to rising burden of dengue and the industry’s attempt to develop vaccines and treatment for dengue,^21 23 24^ it posits an important area for further exploration from a resource allocation point of view. Likewise, a differential interest of public funding and industry funding to sponsor scientific research may be a key influencer of global or country health agendas and is a subject for further research.

### Proposed framework for structured comparisons of agenda status

As a culmination of the multistage process of conceptualising arenas and measurements and testing of the process of measurement and comparison on four global health issues, we have proposed a measurement framework for conducting a structured assessment of the agenda status of global health issues in table 1. Along with the arenas and plausible measures under each arena, we have suggested the sources for data and indicators relevant to each measure tested in our study. Additionally, we have included some measures not used in our analysis (dropped during the stage of screening the indicators) but may have comparative data available at global or national levels. This framework intends to help analysts use feasible and valid indicators (not necessarily all the indicators listed here) in the context of health issues and countries.

**Table 1:** Framework for comparative assessment of agenda status of global health issues

Arenas	Measures	Data sources	Indicators
International aid arena	Development assistance for health (DAH)—dollars allocated and dollars spent	Global Health Visualization tool (Institute for Health Metrics and Evaluation) World Health Financing Report	Annual amount allocated in US dollars for each condition (DAH). DAH as the proportion of total spending on the health condition.
Pharmaceutical industry arena	Clinical trials (industry sponsored)	ClinicalTrials.gov	Number of industry-sponsored clinical trials. Proportion of industry-sponsored clinical trials out of total trials.
Scientific research arena	All registered clinical trials and not industry-sponsored trials	WHO Clinical Trials Registry and ClinicalTrials.gov	Number of trials registered (all stages). Proportion of non-industry-sponsored clinical trials out of total trials.
	Bibliographic trends	PubMed, *The New England Journal of Medicine*, ScienceDirect	Number of publications that included the health issue.
	Systematic reviews registered*	PROSPERO	Number of protocols registered that included the given health issue.
	Series publications funded (such as *The Lancet*)	*The Lancet*	Number of series commissioned/published on a topic.
News media arena	News publishing trends	Access World News database, Vanderbilt Television News Archive	Number of news items listed per topic.
	Google News Archives*	Google News	Number of news items listed per topic.
	Topic search trends on internet*	Google Trends: global search trends broken down by date range, geolocation, category, search type	Number of times a condition appears in searches.
International representation arena	Relevant SDG indicator progress*	UN-DESA SDG implementation data or WHO Global Health Observatory data	Coverage/progress on data against relevant SDG targets, subtargets and disease-specific indicator.
	Resolutions adopted by global health governing bodies such as UNGA and WHA	WHO and UNGA websites, Google searches	Number of resolutions related to each health condition.
	Global health (or national level) partnerships/commissions/High level expert groups (HLEG)/treaties formed on the issue†	Websites of key global health bodies, social media handles	Number of partnerships/treaties/conventions initiated by GH bodies.
Civil society arena	Fundraising† or programmatic activity among non-state organisations (in global or country contexts)	CSO websites	Number of programme activities related to each disease condition.

* Results on these indicators are not presented in this paper but can be useful for further analyses.

† Data for these indicators were not accessible/comparable for our global analysis, but the same can be possible for country-level analyses.

CSO, civil society organisation; GH, global health; SDG, Sustainable Development Goal; UN-DESA SDG, United Nations Department of Economic and Social Affairs Division for Sustainable Development Goals; UNGA, United Nations General Assembly; WHA, World Health Assembly.

This work has advanced the understanding of some aspects of global health agendas, which were outlined for further exploration in the earlier paper. The arenas and indicators presented in this work are, in a way, mediators of various global health activities that in an ideal world would ultimately lead to a reduced burden of specific diseases/health issues. Based on this exercise and the unavailability of comparable measures and data sets, we believe that identifying new transnational arenas and their relevant markers of agenda priority would be challenging. Several new national arenas, however, could be identified and integrated into the model and may offer conceptual advancements to the model and actionable insights to rationalise resource allocations for health issues. For instance, country data could be more readily available for country governments and ministries of health, local civil society, health service providers, professional health associations and policy-programme communities, among other possibilities for national arenas. We have explored a few aspects in a separate paper,^10^ where we have discussed the agenda priority for some disease conditions in relation to policy theories, such as punctuated equilibrium and rational and socially constructed models of policy priorities. This paper offers insights into issues of what magnitude of change is normal and what is reflective of major punctuations. However, many other remaining aspects suggested in the first paper are yet to be further developed and debated.

We acknowledge certain limitations to our approach, which need further testing, finding additional measures and more conceptual-operational detailing. For example, the civil society arenas and international representation arena may be further developed by looking at additional comparable sets of actors and their resource allocation markers to health issues globally, regionally or at country levels. The analysis based on the global civil society arena remains most limiting because of the unavailability of data sets on resource allocations by CSOs. Additionally, there are several interactions and influences among the arenas, and some arenas could be the leaders in agenda setting and others can be followers for different disease conditions. For example, higher international aid and a higher global priority for TB could be linked to higher country-level priority and resource allocations to TB, which may reflect the higher representation of TB in scientific research publications. However, it was not feasible to tease out these interactions in this work, and doing so may require additional conceptualisation and research. The current analysis is also limited by the nature and availability of valid, comparable and quantifiable measures for each arena to establish the agenda status of various health conditions. The data used for our analysis have been sourced from various data sets, all originally produced for other purposes. This itself presents a limitation of appropriateness and comparability on many occasions. For instance, one of the challenges of using the DAH database is that we are limited by it because not all disease conditions are presented separately. To cite an example, one disease of interest, dengue, is clubbed under NTDs, which in turn is clubbed under ‘other infectious diseases’. This makes data mining harder and reveals the invisibility of certain diseases over others, making duplication and use of this framework harder for future research. Further, while our analysis was global, we are mindful that regional/national analyses may reveal a different picture of prioritisation as well as the dominance of certain arenas over others. For example, in a national-level analysis, the role of the government, the Ministry of Health and its policy levers are important to consider since national-level disease priorities are often directly dependent on the government’s attention and political interests. While including this as an arena in the current analysis was discussed among the authors, it was difficult to create a comparable and quantifiable measure for it for the purposes of a global analysis.

Moreover, it is logical that as the incidence of a particular disease condition reduces, its prioritisation in the global health agenda should be reduced. However, this was difficult to establish and compare in the scope of our current analysis. It is also worth noting that this approach offers a descriptive comparison of health issues receiving priority in any defined period and helps visualise the trends in the rise or fall of global health issues on the agenda. However, this approach does not necessarily explain ‘why’ or ‘how’ certain issues get prioritised or neglected in global health, which has been discussed in the political prioritisation and agenda-setting literature^724^,^32^ and is the subject of another study that is part of the GHAP. Nonetheless, the possibility of analysing trends in the agenda status offers better scope for examining ‘why’ certain issues get attention or face neglect in certain periods.

## Conclusion

The varying priority for health issues in different arenas, which only occasionally followed the order of the global burden of studied diseases, reaffirms the political and complex nature of agenda setting and may indicate the differential power and interests of global health actors linked with health issues. At the same time, a close association of disease priorities in most arenas with the international development goals indicates a major influence of the development agendas on priorities received by specific health issues in other arenas. The expanded framework offered in this paper can bring much-desired objectivity in understanding the agenda status of any health issues. This paper’s demonstration of trend analysis can help analysts understand how attention to global health issues changes over time. Additionally, such analyses can offer important insights to the global health community and policymakers in country settings about the interests and priorities of various influential actors. Ultimately, we hope that more objective measurements of health issue prioritisation by different actors can help move beyond the impressionistic claims of priorities and contribute to a more just allocation of resources to global or national health issues.

This framework could have several practical applications. With the advancement of priority measurements in transnational and national arenas, it may be possible to redirect resources in various arenas to critical yet neglected health issues globally. It may also be possible for global health advocates to engage with actors in multiple arenas and understand what it may take to engage them towards improving equity in global health efforts. These measurements can also inform evolving global health commitments and frameworks like resource allocation for global health security, pandemic preparedness and emerging health issues with climate-related threats. Likewise, nationally, a more just allocation of resources by different influential actors may be possible. For instance, a higher resource allocation by the pharmaceutical industry or scientific research arena to a disease condition with a relatively low burden in a particular country may indicate a higher global influence on local health agendas. This framework and analysis can also help policymakers direct resources towards locally prevailing diseases in line with contextual needs, compared with focusing on globally influenced priorities.

## Supplementary material

10.1136/bmjgh-2023-014884 online supplemental file 1

## Data Availability

All data relevant to the study are included in the article or uploaded as supplementary information.
