# Supplementary material for: Comparing priority received by global health issues: a measurement framework applied to tuberculosis, malaria, diarrhoeal diseases and dengue fever
Source: BMJ Glob Health. 2024 Jul 8;9(7):e014884. doi: 10.1136/bmjgh-2023-014884 (PMC11256119; doi:10.1136/bmjgh-2023-014884)

Supplementary file for "Comparing the priority received by global health issues: a measurement framework applied to tuberculosis, malaria, diarrhoeal diseases, and dengue fever"

Supplementary Table: Preliminary listing for arenas, measures, indicators, and potential data sources with discarded measures highlighted in grey.

Arena	Measure	Indicators	Data sources
**International aid**	Development Assistance For Health Financing ($ spent)	$ committed and $ spent	Global Health Visualization tool (Institute for Health Metrics and Evaluation, 2019)
	Inclusion in priority/strategy of international aid	Number of strategic roadmaps (planning/ commitments) from major donors that mentioned the condition	Websites (annual reports, strategy/vision documents/commitments announced)
	Availability of coverage/outcomes/impact data	Narrative/ qualitative	Website, periodic publications
	availability of cost-effectiveness studies/evaluated interventions	Narrative/ qualitative	Article/Journal databases (such as PubMed)
**Pharma industry**	Clinical trials (industry-sponsored)	Number of trials	The Australian New Zealand Clinical Trials Registry, WHO Clinical Trials Registry and ClinicalTrials.gov
	Publications by Big Pharma (clinical and non-clinical)	Number of publications	Web of Science
**Scientific research**	Clinical trials (not industry-sponsored)	Number of trials	The Australian New Zealand Clinical Trials Registry, WHO Clinical Trials Registry and ClinicalTrials.gov
	Bibliographic trends	Number of publications	PubMed; The New England Journal of Medicine; The Lancet (Science Direct)
	Systematic reviews registered	Number of reviews registered	PROSPERO
	Series publications	Number of series published	Lancet

**News media**	Google News trends	Number of news articles that covered (mentioned at least once) a disease condition	Google news database
	News publishing trends	Number of news pieces covering the disease condition (mentioning at least once)	Access World News database; Vanderbilt Television News Archive, Factiva news database
	Topic search trends to Internet-based readership and interest	Number of mentions of the topic	Google Trends
	Twitter trends	Number of tweets where an issue is mentioned	Twitter Analytics
**Civil society**	Fundraising or programmatic activity among non-state organisations in official relations with WHO in 2020	Number of program activities related to each disease condition	CSO Websites (Wayback Machine)
	CSO Partnerships/taskforce/advocacy networks/activist movements declared specific to a condition	Number of partnerships formed/active/met	CSO websites
	Service user/community-based platforms created on the issue	Number of platforms	CSO websites
	Public advocacy/announcements/statements/engagements on the issue	Number of months/quarters/years when the issue was covered or discussed at least once	Social media feed of CSOs
**International representation**	Global health partnerships/commissions/HLEG/treaties formed on the issue	Number of partnerships/treaties/conventions initiated by GH bodies, which may or may not have govt participation	Websites of key global health bodies, social media handles
	Pledges, goals, and commitments announced globally by GHG bodies	Number of publicly available pledges/ goals/commitments	Websites of key global health bodies, social media handles
	Relevant SDG indicator progress	Coverage/progress on indicators against relevant SDG targets	UN-DESA SDG implémentation data

	Resolutions endorsed by major global health governing bodies	Number of resolutions related to each disease condition	WHA and UNGA resolutions from websites

Supplementary Figure 1: Global mortality trends by disease, 2000-2019 (GBD estimates)

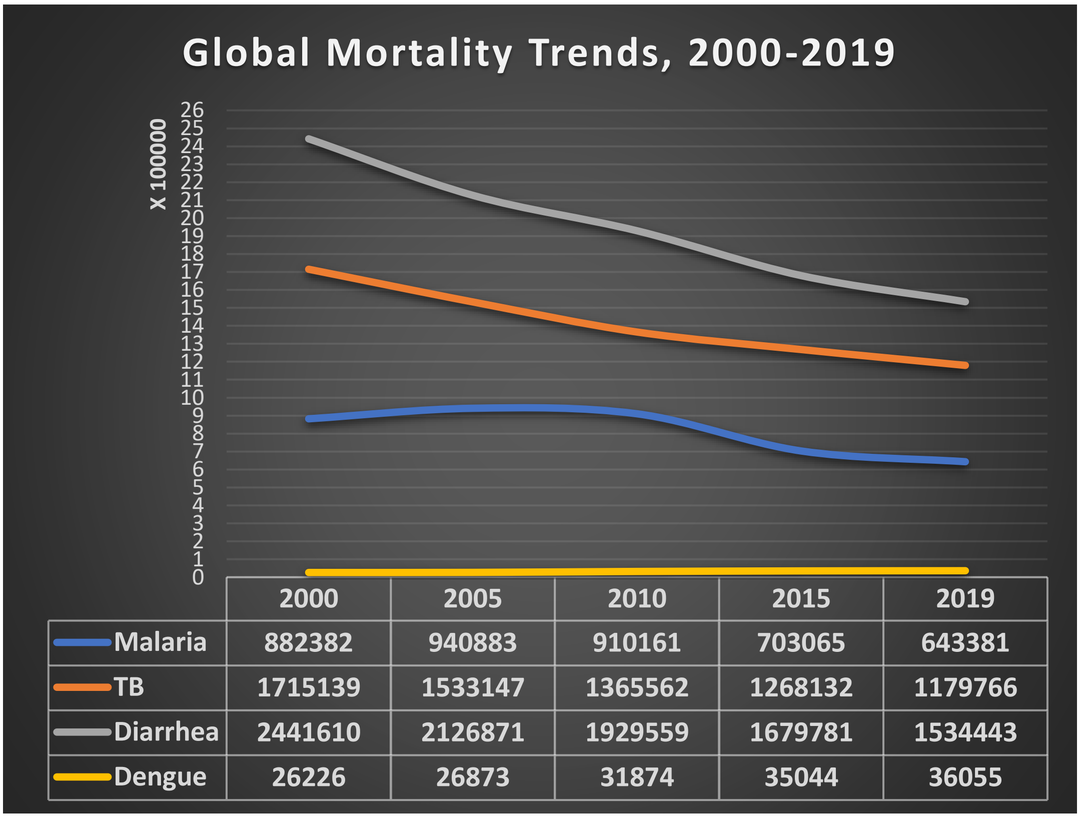

Supplementary Figure 2: **All trials and non-industry-sponsored trials from ClinicalTrials.Gov (2000-2022)**

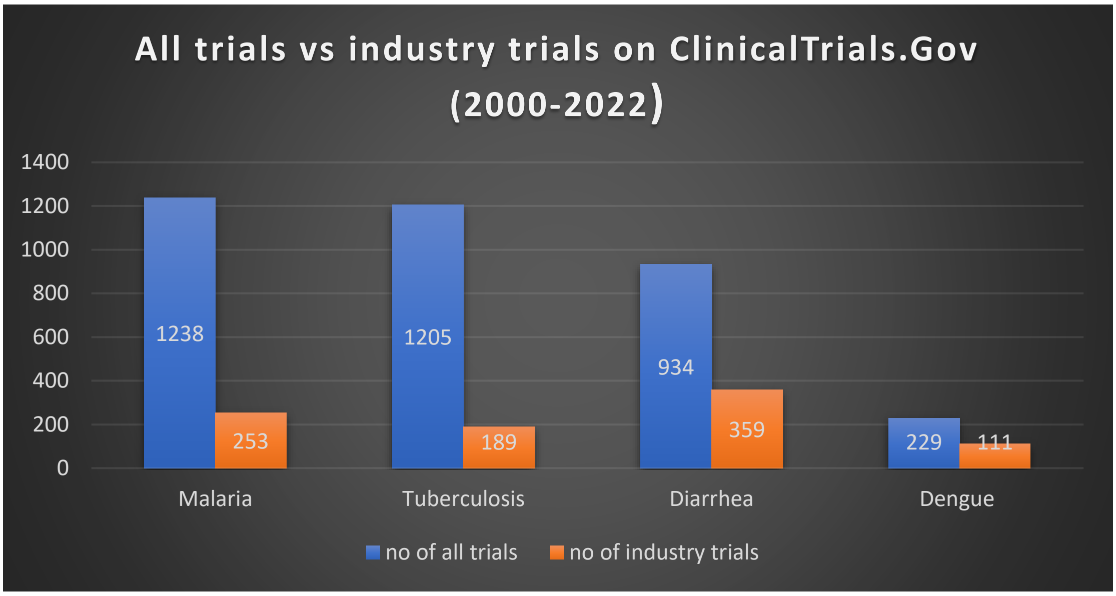

Supplementary Figure 3: Annual number of UNGA resolutions for each condition (2000-2021)

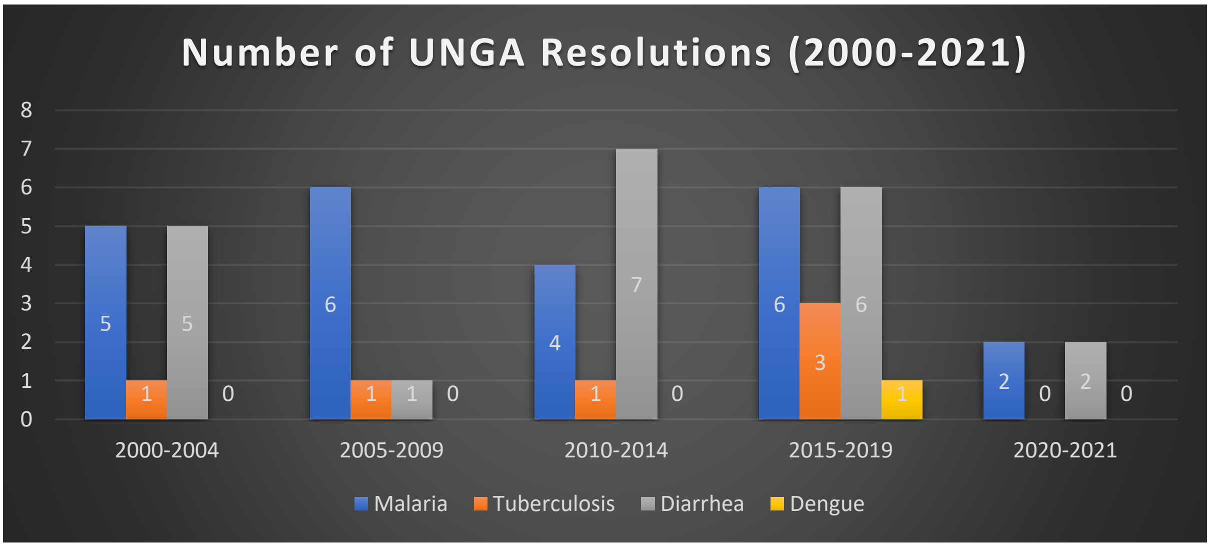

Supplementary Figure 4: CSO program activities related to each condition (2018-2021)

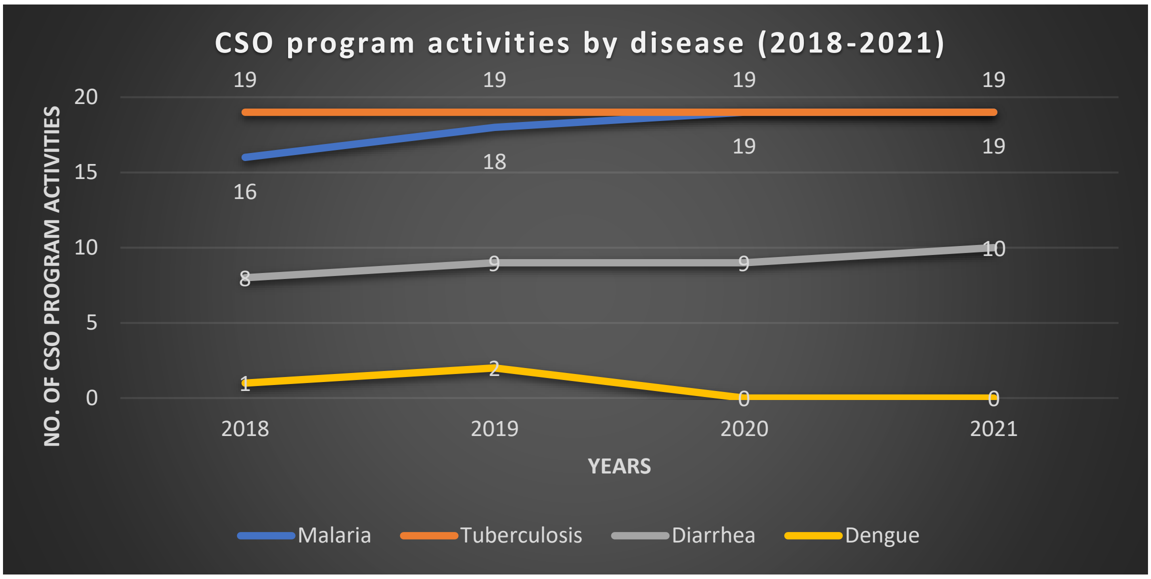

Supplement: online supplemental file 1 [file bmjgh-2023-014884supp001.pdf]
